# DNA methylation changes in Medicago sativa under salt-alkaline stress and the function of 5-azacytidine in enhancing stress tolerance

**DOI:** 10.1186/s12870-025-07424-7

**Published:** 2025-10-21

**Authors:** Rong Gao, Fenqi Chen, Lijuan Chen, Huiling Ma

**Affiliations:** College of Pratacultural Science, Gansu Agricultural University, Yingmen Village, Anning District, Lanzhou, 730070 China

**Keywords:** Salt-alkali stress, DNA methylation, Alfalfa, 5-azacytidine, Food security

## Abstract

Salt-alkali soils severely affect the yield and quality of alfalfa (*Medicago sativa*), yet the epigenetic mechanisms underlying salt-alkali tolerance remain poorly understood in forage plants. Here, we integrate transcriptome profiling with a chemical demethylation approach to reveal how 5-azacytidine (5-AzaC) remodels the methylome and enhances stress performance. Our research revealed that salt-alkali stress induced a decrease in methylation levels at CG and CHG sites, while methylation at CHH sites increased, with notable differences observed particularly in promoter and transposable element regions. The DNA methyltransferase inhibitor 5-azacytidine (5-AzaC) enhanced alfalfa’s tolerance to salt-alkali stress. Transcriptome analysis showed that this was associated with upregulation of genes related to phenylpropanoid metabolism, flavonoid biosynthesis, and glutathione metabolism. McrBC-PCR confirmed the presence of DNA methylation modifications in the promoter regions of key salt-alkali response genes *4CL*, *GCL*, and *HCT*, and these genes were found to potentially interact with transcription factors such as *NAC*, *bHLH*, and *ERF*. These findings highlight the role of epigenetic regulation in alfalfa’s salt-alkali tolerance, providing theoretical guidance for breeding resilient forage cultivars.

**Supplementary Information:**

The online version contains supplementary material available at 10.1186/s12870-025-07424-7.

## Introduction

 Soil salinization has emerged as a critical global issue threatening agricultural productivity, environmental sustainability, and ultimately, food security [1]. Currently, saline soils cover an estimated 1.381 billion hectares, accounting for 10.7% of the Earth’s terrestrial surface. With ongoing global warming, this area is projected to expand to 24–32% of the global land surface by the end of the century [2]. Saline-alkaline stress severely hampers plant growth and significantly reduces crop yield, nutritional quality, and economic value. In the context of rising global food demand, developing effective strategies to improve crop tolerance to salinity and alkalinity has become an urgent priority. Although conventional breeding has contributed to enhancing stress tolerance, its limitations in efficiency and adaptability have led to growing interest in epigenetic mechanisms, as promising alternative regulatory strategies [3]. Among these, DNA methylation has emerged as a promising regulatory layer that complements traditional approaches. Recent studies have demonstrated that DNA methylation plays a key role in plant adaptation to environmental stresses, with strong associations to stress tolerance, yield stability, and quality traits [4, 5]. Understanding the function of DNA methylation in stress responses is thus essential for developing climate-resilient, high-quality crops, including forage plants critical to livestock nutrition and food system sustainability.

DNA methylation refers to the process by which a methyl group is transferred to specific bases by DNA methyltransferases. It primarily occurs at cytosine residues in CG, CHG, and CHH (H = A, C, or T) contexts, and plays a critical role in gene expression regulation and genome stability maintenance [6]. Studies have shown that dynamic changes in DNA methylation are involved in various aspects of plant growth and development, including seed germination, flowering, and fruit ripening, as well as in responses to abiotic stresses such as salinity, heavy metals, drought, and low temperature [7, 8]. DNA methylation is closely associated with plant stress tolerance, especially through its effects on the methylation status of promoters, transposable elements (TEs), and gene bodies, which in turn can modulate the expression of stress-responsive genes [9]. For example, treatment with DNA methylation inhibitors has been shown to significantly enhance stress resistance in tomato [10], rice [11], and soybean [4], suggesting that dynamic changes in DNA methylation may play an important role in plant adaptation to adverse environments. In rice, tetraploid lines exhibit stronger salt tolerance compared to diploids, a phenomenon attributed to polyploidy-induced CHH-type DNA hypomethylation [12]. Similarly, in tomato, SlSAMS1 mediates elevated methylation levels within the gene body of SlGI, leading to activation of downstream genes and enhanced salt tolerance [13]. In maize, ZmKTF1 regulates CHH methylation in the rboh gene region through an RNA-directed DNA methylation (RdDM) pathway, thereby maintaining reactive oxygen species (ROS) homeostasis and improving salt tolerance [14]. Moreover, environmental factors can induce dynamic changes in DNA methylation that further regulate gene expression. For instance, under high humidity stress, CHH demethylation occurs in the promoter regions of ACS1 and ETR1 in Arabidopsis, resulting in their upregulated expression and increased ethylene production [15]. In rice, CHH hypomethylation in the ACT1 promoter enhances binding of the transcription factor Dof1, significantly increasing ACT1 expression and promoting plant adaptation to cold environments at high latitudes [16].

Soil salinization and alkalization are major environmental factors limiting the cultivation of Medicago sativa, significantly impairing its growth, yield, and forage quality [17]. As a high-protein forage crop widely used in animal husbandry, the quality and productivity of alfalfa directly affect the safety and sustainability of livestock-derived food products. However, current research on alfalfa’s response to salt-alkali stress has primarily focused on gene expression and transcriptional regulation, while the contribution of epigenetic mechanisms—particularly DNA methylation-remains largely unexplored. This study aims to elucidate genome-wide DNA methylation dynamics in alfalfa under salt-alkali stress and to investigate the role and mechanism of the DNA methyltransferase inhibitor 5-azacytidine (5-AzaC) in enhancing stress tolerance. Our findings provide novel insights into the epigenetic regulation of alfalfa’s response to salt-alkali conditions. This study not only advances the molecular understanding of alfalfa’s adaptation to saline-alkaline environments but also lays a theoretical foundation for the development of stress-resilient, high-quality forage cultivars.

## Materials and methods

### Plant material and treatment

Cultivar alfalfa ‘Gannong NO.3’ was used in this study. Selected uniformly full alfalfa seeds were soaked in a 6% sodium hypochlorite solution for 10 min and then rinsed three times with distilled water. These seeds were sown in planting pots filled with vermiculite under controlled growth conditions with a photoperiod of 16/8 h (light/dark), a light intensity of 200 µmol m^−2^ s^−1^, and a temperature of 25 °C. Four days after sowing, alfalfa seedlings were transplanted into 1/2 Hoagland nutrient solution for hydroponics. When the seedlings reached 4 weeks of age, they were divided into control and experimental groups for 5 d pre-treatment period in the nutrient solution, with or without the addition of 100 µM 5-AzaC. After the pre-treatment, the experimental group was subjected to salt-alkaline treatment (75 mM solution with Na₂CO₃ and NaHCO₃ mixed at a 1:2 molar ratio of Na⁺ ions, pH approximately 9.3). Leaves from both Saline-Alkaline -treated and untreated plants were harvested after 48 h immediately frozen in liquid nitrogen. These samples were subsequently used for RNA-seq analyses. After 7 days of treatment, samples were collected for physiological measurements.

### Determination of agronomic characters

Plant height, fresh weight (FW), and dry weight (DW) of alfalfa shoots were measured. For FW, the surface of the shoots was dried before weighing. For DW, samples were first inactivated at 105 °C for 15 min, then dried in an oven at 80 °C until the weight remained constant [18].

### Determination of pigment, crude protein, and acid detergent fiber (ADF) contents

Arnon’s method [19] was used to determine the total chlorophyll contents. 0.1 g of leaf tissue was leached in 10 ml of an 80% acetone solution. After 48 h, the absorbance values of the extraction solution at 663 nm, 645 nm, and 480 nm were measured using a spectrophotometer.

Crude protein content was measured by the Kjeldahl method, which involves digestion, distillation, and titration to determine total nitrogen content, subsequently converted to protein content using a conversion factor of 6.25 [20].

Acid detergent fiber (ADF) content was determined as follows. Approximately 2 g of dried leaf sample was treated with acid detergent solution and boiled for 1 h. The residue was then washed four times with warm water, twice with distilled water, and once with acetone, followed by gentle rubbing for 30 min. The sample was subsequently air-dried and weighed to constant weight to calculate ADF content [21].

### Leaves malondialdehyde (MDA) content and proline quantification

Determination of malondialdehyde (MDA) content: Fresh alfalfa leaves (0.3 g) were homogenized in 0.1% trichloroacetic acid (TCA) and centrifuged at 10,000 rpm for 15 min. The supernatant was mixed with 0.5% thiobarbituric acid (TBA) dissolved in 20% TCA, then heated at 95 °C for 30 min and rapidly cooled in an ice bath. After centrifugation, the absorbance of the supernatant was measured at 532 nm and 600 nm.

Determination of proline content: 0.3 g of fresh leaf tissue was homogenized in 5 mL of 3% sulfosalicylic acid and centrifuged at 10,000 × g for 10 min. Two milliliters of the supernatant were mixed with 2 mL of acid ninhydrin reagent and 2 mL of glacial acetic acid. The mixture was incubated in a boiling water bath for 30 min, then rapidly cooled in an ice bath to terminate the reaction. The chromophore was extracted with 4 mL of toluene, and its absorbance was measured at 520 nm using a spectrophotometer.

### Measurement of relative electrolyte leakage

1 g of alfalfa leaf samples was collected, veins removed, and leaves cut into small pieces. The leaf pieces were soaked in 20 mL of distilled water for 12 h. After soaking, the initial conductivity (EC1) of the solution was measured. The samples were then heated in a 100 °C water bath for 30 min to obtain the final conductivity (EC2). Relative electrolyte leakage was calculated as (EC1/EC2) × 100%.

### Quantification and visualization of reactive oxygen species (ROS)

The hydrogen peroxide (H_2_O_2_) and superoxide anion radical (O_2_^•−^) content were assayed with a fluorescent dye 2,7-dichlorofluorescin diacetate and the sulphanilic acid and α -naphthylamine solution as described by Shi et al. [22].

### Measurements of antioxidant enzyme activity

Grind 0.3 g of fresh alfalfa leaves into a homogenate with 5 mL of 50 mM potassium dihydrogen phosphate buffer (pH 7.8) containing 0.1 mM EDTA. Centrifuge at 12,000 g for 20 min. The activities of SOD, POD, CAT, and APX were determined according to the method of Sheikh Mohammadi et al. [23].

### Transcriptome sequencing (RNA-seq) and data processing

Total RNA was extracted from alfalfa leaves using TRIzol reagent (Tiangen, China). RNA integrity and concentration were assessed with an Agilent 2100 Bioanalyzer. Poly(A) mRNA was enriched using Oligo(dT) magnetic beads and subsequently fragmented by divalent cations. The fragmented mRNA was reverse-transcribed to synthesize first-strand cDNA, followed by second-strand synthesis with DNA polymerase I. The cDNA fragments then underwent end repair, addition of A-tails, and ligation of sequencing adapters. Fragments between 370 and 420 bp were size-selected and PCR-amplified to construct the sequencing library. Following quality control, libraries were sequenced on the Illumina platform to generate 150 bp paired-end reads.

Raw sequencing reads were processed with Trim_galore to remove adapters and low-quality bases, yielding high-quality clean reads. Clean RNA-seq reads were aligned to the *Medicago sativa* reference genome (XinJiangDaYe cultivar, available at https://modms.lzu.edu.cn/alfalfa/download/downloadFile?name=XJDY&suffix=genome) using HISAT2 v2.0.5 with default parameters. Gene expression levels were quantified as fragments per kilobase of transcript per million mapped reads (FPKM). Genes with FPKM ≥ 1 were retained for downstream analyses. Differentially expressed genes (DEGs) were identified using DESeq2 [24] with criteria of fold change ≥ 1 and adjusted p-value ≤ 0.05. KEGG pathway enrichment analysis was conducted using clusterProfiler (v3.8.1), with pathways considered significantly enriched at adjusted *p* < 0.05 [25].

### Whole-genome bisulfite sequencing (WGBS) and data analysis

Raw sequencing data were processed for quality control using FastQC and fastp to generate clean reads. Clean reads were mapped to the Medicago sativa reference genome (cultivar XinJiangDaYe, https://modms.lzu.edu.cn/alfalfa/download/downloadFile?name=XJDY&suffix=genome) using Bismark (v0.24.0), and duplicates were removed. Cytosines with at least three reads were included for further analysis. Methylation levels were calculated as the ratio of methylated cytosines (mC) to total cytosines (mC + nonmC). Differentially methylated regions (DMRs) were identified using DSS (Differentially Methylated Sites) with FDR < 0.05 and methylation differences greater than 0.1 [26].

### Quantitative RT-­PCR (qRT-PCR)

Total RNA from plants was extracted using TRIzol reagent (Tiangen, Beijing, China). Subsequently, cDNA was synthesized using the PrimeScript™ II 1 st Strand cDNA Synthesis Kit (Takara, Dalian, China). qRT PCR was carried out using the LightCycler 480 Real-Time PCR System (Roche Applied Science) and SYBR^®^ Green Premix Pro Taq HS qPCR Kit. The reaction system is 2 × SYBR Green Pro Taq HS Premix 10 µL, Primer F 0.4 µL, Primer R 0.4 µL, cDNA 2 µL, ddH_2_O 7.2 µL. PCR reaction conditions: 95 °C, 30 s; 95 °C, 5 s, 60 °C, 30 s, 40 cycles; 4 °C, ∞. All data were normalized to the expression of Actin gene (MS.gene049321.t1), and relative expression levels were calculated using the 2^−ΔΔCt^ method. Primers for qRT-PCR are listed in Table S1.

### McrBC-PCR assay

The methylation status of specific genomic regions was evaluated using the McrBC-PCR assay. Genomic DNA was isolated from alfalfa seedling leaves using a plant genomic DNA extraction kit (Tiangen, Beijing). For each sample, 1 µg of DNA was digested with McrBC enzyme (Takara, Japan) in the presence of guanosine-5’-triphosphate (GTP), following the manufacturer’s instructions. A negative control reaction was performed by replacing GTP with water. The digested DNA served as the template for PCR amplification targeting various promoter regions. PCR products were resolved on a 1% agarose gel. Primer sequences used for McrBC-PCR are provided in Table S1.

### Statistical analysis

Statistical analysis was performed using SPSS 20.0 statistical software (SPSS Inc., Chicago, IL, USA). Values were means ± standard error (SE) of 3 biological replicates. Multiple comparisons were processed by application of Duncan’s multiple range test to determine the significance of the results among different treatments at a *p* < 0.05 level.

## Results

### Salt-alkali stress inhibits alfalfa growth and alters DNA methylation patterns

**Fig. 1:**
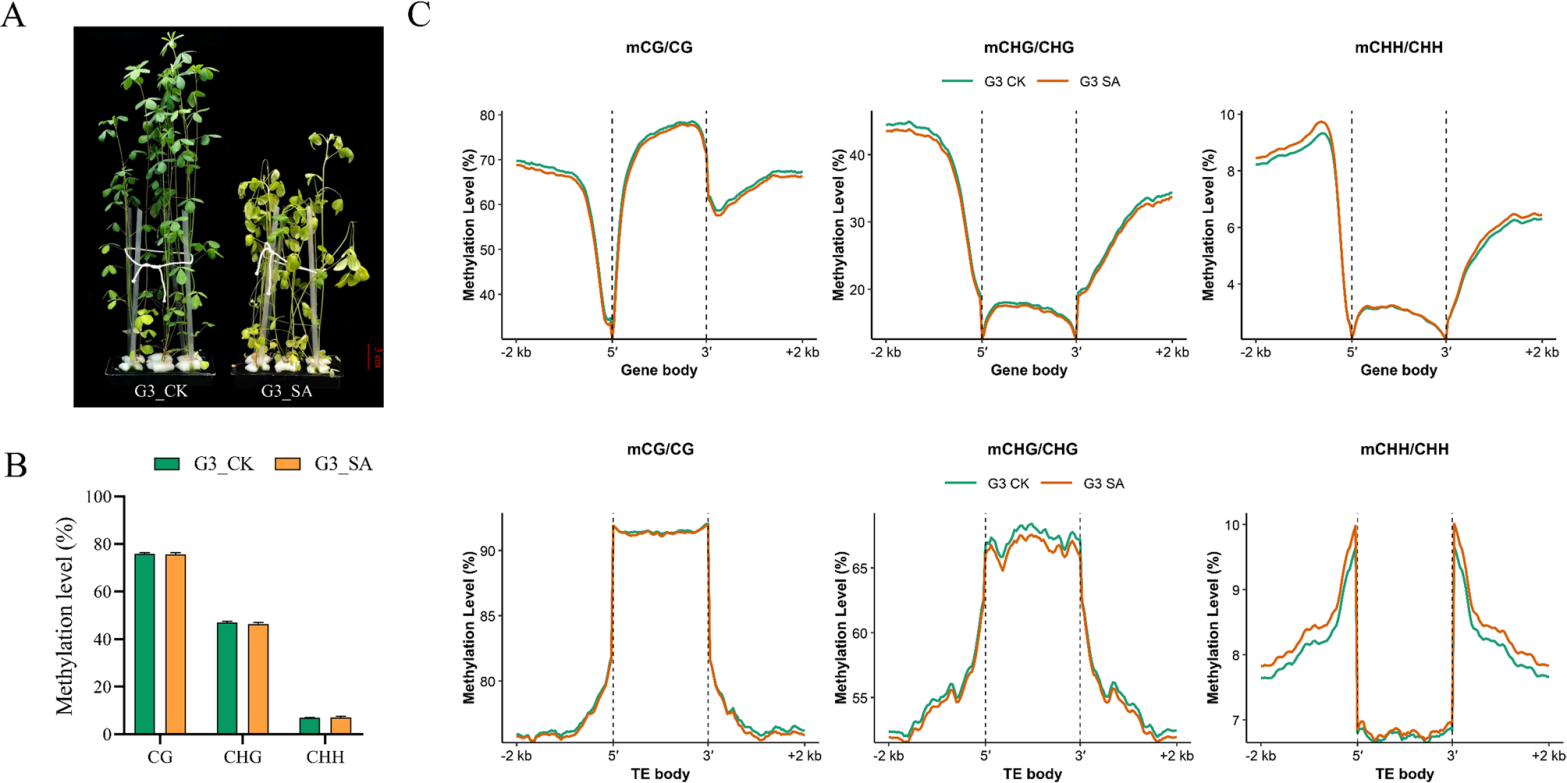
Salt-alkali stress inhibits alfalfa growth and alters DNA methylation patterns. **A** Phenotypic comparison of alfalfa plants under control and salt-alkali stress conditions. **B** Global DNA methylation levels in CG, CHG, and CHH contexts under control and salt-alkali treatment. Data are presented as mean ± SE (*n* = 3). Differences between CK and SA were analyzed by independent-samples *t*-test. **C **and** D** DNA methylation levels in different genomic regions under control and salt-alkali conditions

After 5 d of treatment with 75 mmol L⁻¹ salt-alkali solution, alfalfa growth was significantly inhibited, as indicated by shortened internodes, leaf chlorosis, and premature senescence. The aboveground biomass was markedly reduced compared to the control group (Fig. 1A). Whole-genome DNA methylation analysis revealed a slight decrease in CG methylation levels (from 75.93% to 75.59%) and a substantial reduction in CHG methylation levels (from 76.39% to 47.00%), whereas CHH methylation levels slightly increased (from 6.95% to 7.07%) (Fig. 1B). However, these changes were not statistically significant (*p* > 0.05). Further analysis of differentially methylated regions (DMR) showed that DMRs were predominantly enriched in gene promoter regions and transposable elements (TEs). In promoter regions (2 kb upstream of the transcription start site), both mCG and mCHG methylation levels were reduced under stress conditions, while mCHH methylation levels were elevated (Fig. 1C). In TEs, methylation changes were primarily observed in the mCHG context, with a notable decrease in methylation levels upon stress treatment (Fig. 1D), suggesting differential DNA methylation may participate in the plant’s response to salt-alkali stress by regulating gene expression and maintaining genome stability.

### Application of the DNA methylation inhibitor 5-AzaC improves alfalfa tolerance to salt-alkali stress

**Fig. 2:**
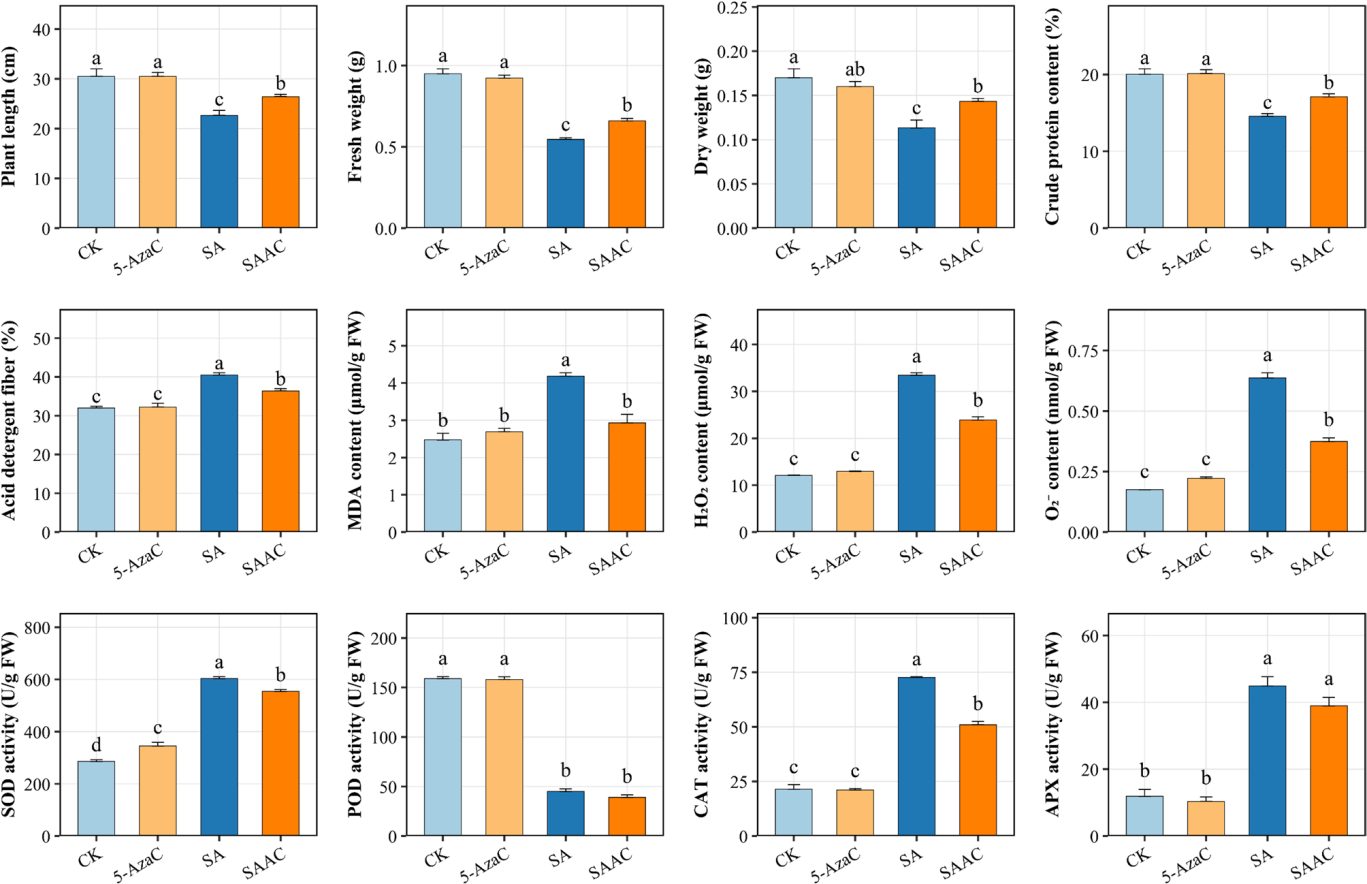
The effect of 5-AzaC pretreatment on alfalfa under salt-alkali stress. Bar plots show the mean ± standard error (SE) of various parameters in alfalfa under four treatments: CK (control), 5-AzaC, SA (salt-alkali), and SAAC (salt-alkali + 5-AzaC). Different lowercase letters indicate statistically significant differences between treatments within each trait according to Duncan’s multiple range test (*p* < 0.05)

Salt-alkali stress decreased pigment content, impaired membrane stability, and induced oxidative damage, resulting in reduced biomass accumulation and quality decline in alfalfa. Notably, pretreatment with 5-AzaC (SAAC) significantly mitigated the physiological damage caused by salt-alkali stress. Compared with the SA treatment, SAAC increased plant height (26.43 cm vs. 22.67 cm), fresh weight (0.66 g vs. 0.55 g), and chlorophyll content (8.40 mg/g, representing a 1.8-fold increase). Moreover, SAAC improved the nutritional quality by increasing crude protein content (17.10% vs. 14.56%) and decreasing acid detergent fiber (ADF) content (36.40% vs. 40.48%). Membrane stability improved, as evidenced by a reduction in relative electrolyte leakage to 67.63%, compared with 56.34% under SA, and a decrease in malondialdehyde (MDA) content from 4.18 to 2.30 µmol/g. Markers of oxidative stress were also alleviated, with hydrogen peroxide (H₂O₂) levels decreasing from 33.44 to 23.89 µmol/g and superoxide anion (O₂⁻) content reduced from 0.64 to 0.37 µmol/g. Concurrently, antioxidant enzyme activities exhibited moderate reductions: superoxide dismutase (SOD) activity declined from 603.22 to 554.07 U/g, while peroxidase (POD) and ascorbate peroxidase (APX) activities showed no significant changes (Fig. 2).

### Transcriptomic analysis of salt-alkali resistance mechanisms induced by 5-AzaC in alfalfa plant

**Fig. 3:**
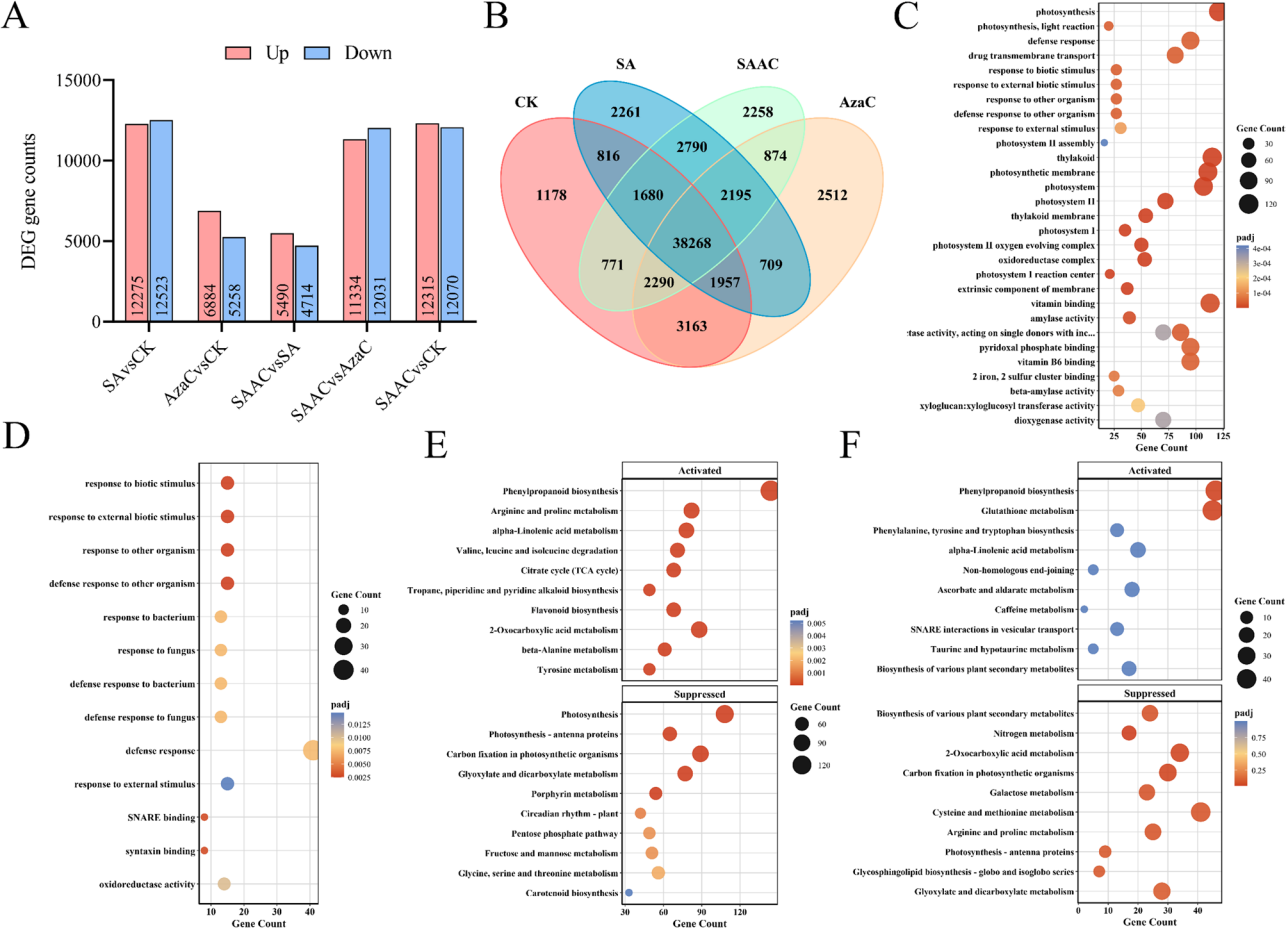
5-AzaC modulates the transcriptomic response of alfalfa under salt-alkali stress. **A** Bar plot showing the numbers of upregulated (red) and downregulated (blue) differentially expressed genes (DEGs) in five pairwise comparisons. **B** Venn diagram illustrating the overlap of DEGs among the four treatment groups: CK (control), 5-AzaC, SA (salt-alkali), and SAAC (salt-alkali + 5-AzaC). **C**, **D** Gene Ontology (GO) enrichment analysis of DEGs in the SA vs. CK comparison (**C**) and SAAC vs. SA comparison (**D**). **E **and** F** KEGG pathway enrichment analysis of DEGs in SA vs. CK (**E**) and SAAC vs. SA (**F**). Enriched pathways are categorized into activated (upper panels) and suppressed (lower panels) groups. Dot size represents the number of DEGs enriched in each pathway, and color indicates the adjusted *p*-value

To elucidate the molecular basis of alfalfa’s response to salt-alkali stress and the regulatory role of 5-AzaC, transcriptome analysis was conducted across five pairwise comparisons among four treatments (Table S2) The principal component analysis (PCA) results showed that the samples from different treatment groups were clearly separated along the first two principal components, indicating significant differences among the treatments (Fig. S1). As shown in Fig. 3A, salt-alkali stress (SA vs. CK) resulted in 13,758 DEGs, including 7,192 upregulated and 6,566 downregulated genes. Under 5-AzaC treatment combined with salt-alkali stress (SAAC vs. SA), 11,154 DEGs were identified, with 5,490 upregulated and 4,714 downregulated. The Venn diagram revealed both shared and unique DEGs between SA and SAAC treatments; 2,195 DEGs were common, representing a conserved stress-responsive core gene set, while SAAC exhibited 1,957 unique DEGs likely linked to enhanced stress mitigation (Fig. 3B).

Gene Ontology (GO) enrichment analysis indicated that in the SA vs. CK comparison (Fig. 3C), DEGs were significantly enriched in photosynthesis-related structures (e.g., photosystem I and II, thylakoid membranes), oxidoreductase and vitamin-binding activities, and biological processes related to responses to external stimuli and defense. In contrast, DEGs in SAAC vs. SA (Fig. 3D) were significantly enriched in oxidoreductase activity and biological processes related to responses to external stimuli.

Kyoto Encyclopedia of Genes and Genomes (KEGG) enrichment showed that SA-induced upregulated genes were enriched in amino acid metabolism (arginine and proline, valine, leucine and isoleucine degradation, tyrosine metabolism), carbon metabolism (citrate cycle), and secondary metabolite biosynthesis (flavonoids, phenylpropanoids, alkaloids) (Fig. 3E). Downregulated genes were enriched in photosynthesis, carbon fixation, photosynthesis antenna proteins, circadian rhythm, carotenoid, and porphyrin metabolism. Compared to SA, SAAC upregulated genes were enriched in glutathione metabolism and phenylpropanoid biosynthesis, implying enhanced antioxidant and defense mechanisms via 5-AzaC. Downregulated genes involved nitrogen metabolism, photosynthesis, and carbon fixation (Fig. 3F).

### The phenylpropanoid and glutathione metabolism pathways are key mechanisms by which 5-AzaC enhances salt-alkali tolerance in alfalfa

**Fig. 4:**
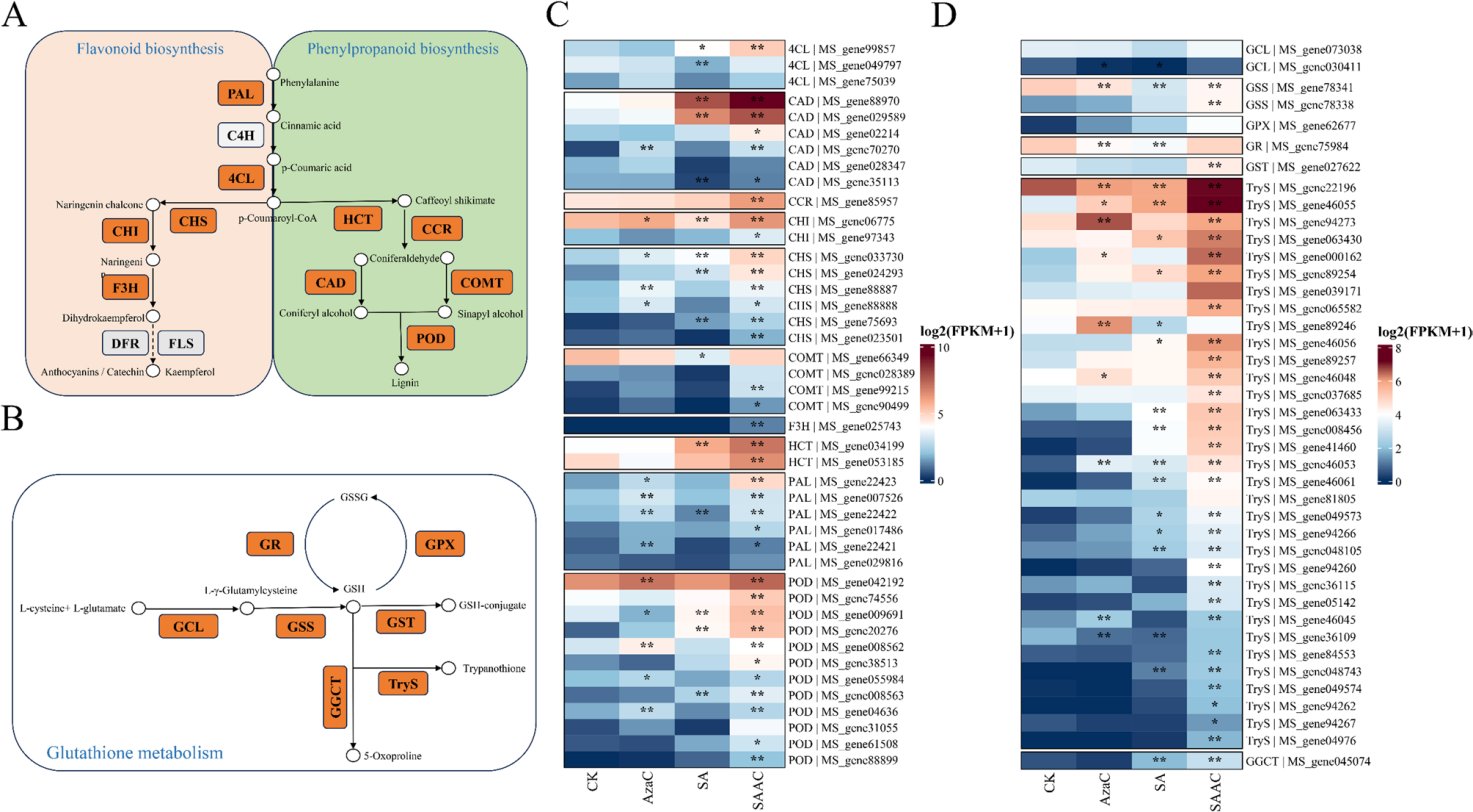
5-AzaC treatment alters the expression patterns of key enzyme genes involved in the phenylpropanoid and glutathione metabolism pathways. **A** Schematic diagram of the phenylpropanoid and flavonoid biosynthesis pathways (KEGG: mtr00940, mtr00941). The orange and green backgrounds represent the flavonoid and phenylpropanoid branches, respectively. **B** Schematic diagram of the glutathione metabolism pathway (KEGG: mtr00480), with orange boxes indicating key enzyme genes included in the expression analysis. **C** Heatmap showing the expression levels of genes involved in the phenylpropanoid and flavonoid biosynthesis pathways under different treatments. **D** Heatmap of gene expression in the glutathione metabolism pathway under different treatments. The color scale represents gene expression levels as log_2_(FPKM + 1). Data are presented as mean ± SE. Significance was assessed using one-way ANOVA, with each treatment compared to the control (CK). *p* < 0.05 (*), *p* < 0.01 (**)

To further investigate the regulatory effects of 5-AzaC treatment on gene expression, we analyzed the expression patterns of key genes involved in the phenylpropanoid and glutathione metabolism pathways (Fig. 4). Figure 4A and B illustrate the main enzymes and metabolic routes of the phenylpropanoid and glutathione pathways, respectively. The heatmap in Fig. 4C shows that key genes in the phenylpropanoid pathway, such as PAL, 4CL, HCT, CCR, CAD, and POD, were significantly upregulated under salt-alkali stress, indicating strong induction compared to the CK. Pre-treatment with 5-AzaC further enhanced the expression of certain genes, particularly CAD and POD, suggesting that 5-AzaC promotes phenylpropanoid metabolism, which may contribute to improved stress resistance.

In the glutathione metabolism pathway (Fig. 4D), salt-alkali stress induced the expression of key genes such as *GSS*, *GGCT*, *GPX*, and *TryS*, and 5-AzaC treatment further elevated their expression levels, indicating an activation of the antioxidant defense system to mitigate oxidative stress. Interestingly, *GCL* and *GST* were suppressed under salt-alkali stress but significantly upregulated following 5-AzaC treatment, implying that 5-AzaC enhances glutathione biosynthesis and detoxification capacity under stress conditions.

### qRT-PCR and McrBC-PCR analysis of key genes Involved in 5-AzaC-induced enhancement of salt-alkali tolerance of alfalfa

**Fig. 5:**
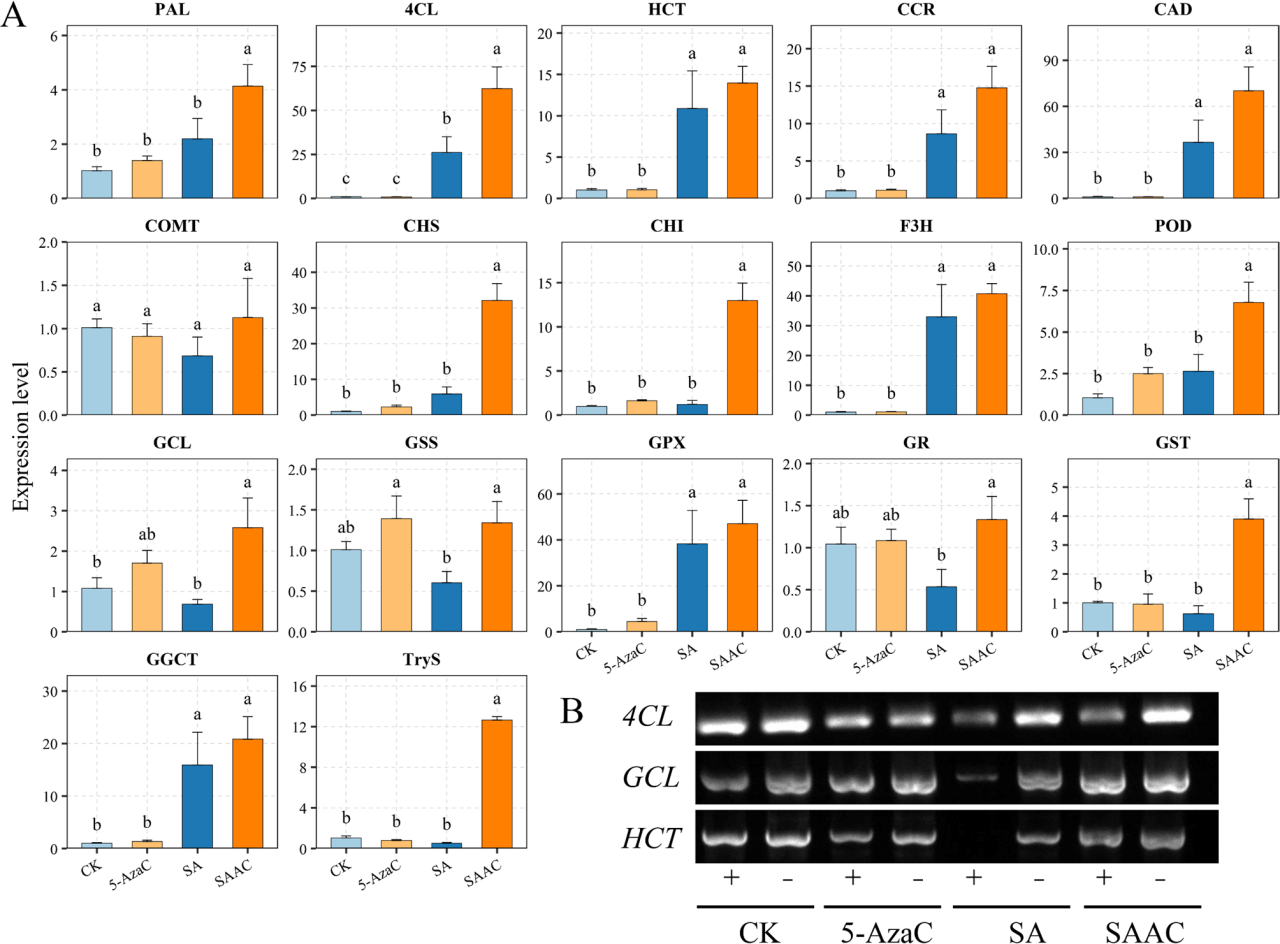
Validation of gene expression and promoter methylation levels of selected genes under CK (control), 5-AzaC, SA (salt-alkali), and SAAC (salt-alkali + 5-AzaC) treatments. (A) qRT-PCR analysis of selected genes under CK, SA, 5-AzaC, and SAAC treatments. Different lowercase letters indicate statistically significant differences among treatments for each gene according to Duncan’s multiple range test (p < 0.05). (B) McrBC-PCR analysis of DNA methylation levels in the 1000-bp upstream promoter regions of selected genes. “+” indicates the addition of GTP (required for McrBC activity), and “–” indicates its absence.

To further investigate transcriptional regulation under salt-alkali stress, we analyzed the expression and promoter DNA methylation of key genes involved in phenylpropanoid and glutathione metabolism. qRT-PCR results showed that salt-alkali stress significantly upregulated *PAL*, *4CL*, *HCT*, *CCR*, and *CAD*. SAAC treatment further enhanced the expression of several genes, particularly *4CL*, *PAL*, and *POD*. Similarly, flavonoid biosynthesis genes (*CHS*, *CHI*) were upregulated under SA and showed even higher expression in SAAC (Fig 5). In contrast, glutathione metabolism genes (*GCL*, *GSS*, *GR*, *GST*) were suppressed by SA but significantly restored in the SAAC group. McrBC-PCR revealed that under CK and 5-AzaC treatments, *4CL*, *GCL*, and *HCT* showed minimal differences between GTP-treated (“+”) and untreated (“–”) samples, suggesting low promoter methylation. Under SA treatment, the “+” band intensity of *4CL*, *GCL*, and *HCT* decreased, indicating increased methylation levels. In the SAAC treatment, the band signals were enhanced, suggesting that the methylation levels decreased compared to SA.

### Co-expression network analysis identifies key transcription factors involved in 5-AzaC induced stress regulation

To further explore the transcription factors (TFs) associated with key metabolic pathways regulated by 5-AzaC, a co-expression network was constructed (Fig. 5). In the network, blue nodes represent TFs, while peripheral-colored circles indicate genes involved in flavonoid biosynthesis (red), glutathione metabolism (orange), and phenylpropanoid biosynthesis (yellow). Co-expression analysis revealed that several TFs, such as *NAC*, *bHLH*, *FAR1*, *ERF*, and *WRKY*, exhibited high connectivity and may play critical roles in regulating these metabolic pathways. Notably, *NAC* and *bHLH* showed the highest number of connections, suggesting they may act as central regulators of 5-AzaC-induced metabolic changes. Moreover, extensive co-expression was observed among genes from different pathways, implying potential crosstalk and coordinated regulation among these stress-responsive metabolic processes.

## Discussion

**Fig. 6:**
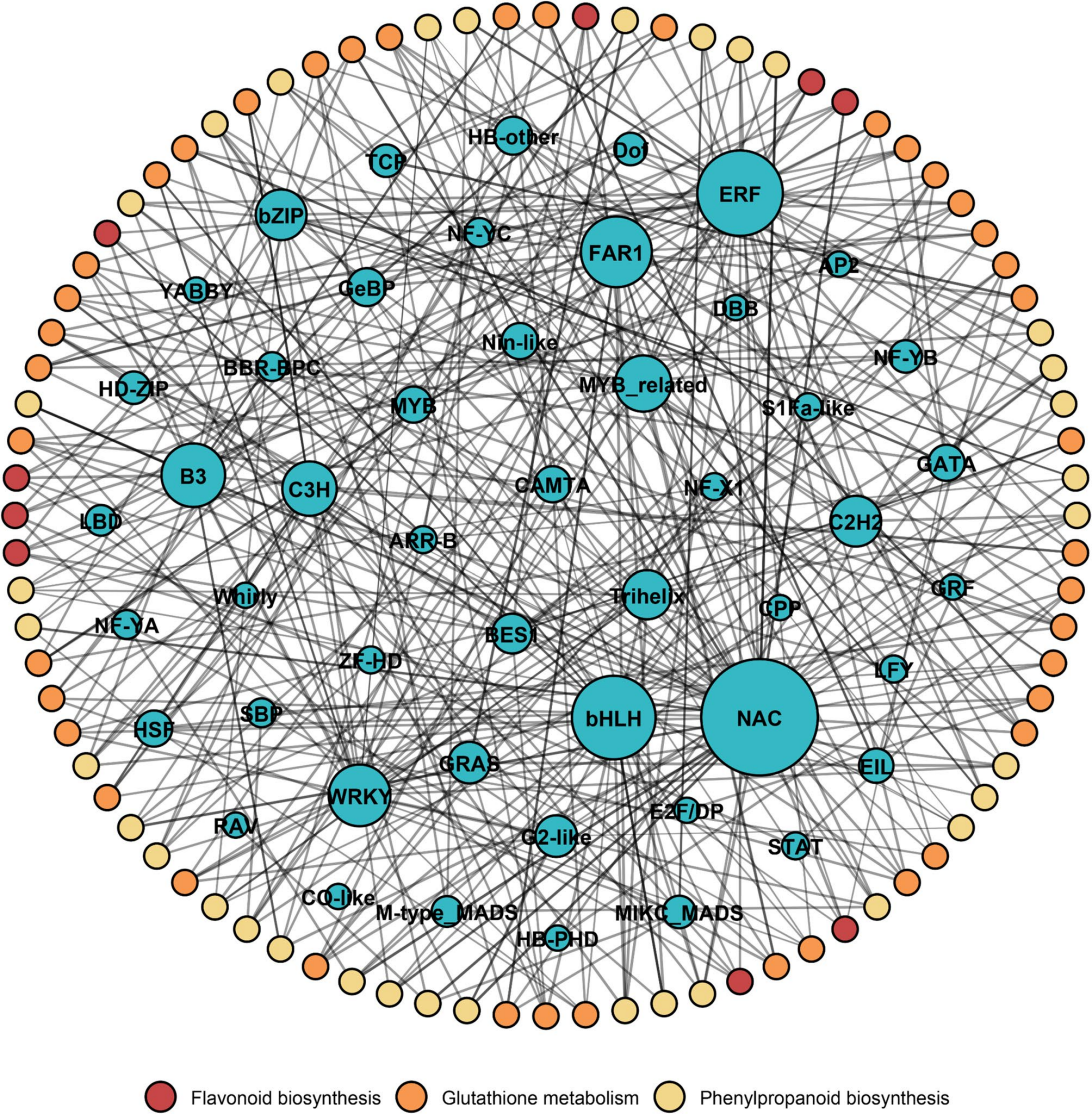
Analysis of transcription factors (TFs) associated with the expression of genes involved in flavonoid, phenylpropanoid, and glutathione metabolism induced by 5-AzaC. The network displays TFs (blue nodes) and genes involved in three key metabolic pathways: flavonoid biosynthesis (red), glutathione metabolism (orange), and phenylpropanoid biosynthesis (yellow). The connecting lines represent potential co-expression relationships between TF and non-TF genes.

Salt-alkali stress is one of the major abiotic factors limiting global agricultural productivity, severely affecting the growth and yield of leguminous forage crops such as *Medicago sativa* [27]. In recent years, DNA methylation, as a key epigenetic regulatory mechanism, has received increasing attention for its role in plant stress responses. In this study, we found that salt-alkali stress induces dynamic changes in genome-wide DNA methylation levels in alfalfa, with differentially methylated regions primarily enriched in promoter and transposable element regions. The application of the DNA methyltransferase inhibitor 5-azacytidine (5-AzaC) significantly enhanced alfalfa’s tolerance to salt-alkali stress while also improving its quality under salt-alkali stress. Transcriptome analysis revealed that this enhanced tolerance was mainly associated with the upregulation of genes involved in key metabolic pathways, including phenylpropanoid metabolism, flavonoid biosynthesis, and glutathione metabolism. McrBC-PCR confirmed the presence of DNA methylation modifications in the promoter regions of these key genes. Combined with protein–protein interaction network analysis, we further found that these genes exhibit coordinated interactions with several major transcription factor families, such as *NAC*, *bHLH*, *ERF*, and *WRKY* (Fig. 6).

Abiotic stresses induce dynamic changes in DNA methylation across the plant genome, serving as a crucial epigenetic regulatory mechanism for plant adaptation to adverse environments [28]. Salt, drought, and low-temperature stresses have all been reported to cause genome-wide alterations in DNA methylation levels [4, 16, 29]. Methylation and demethylation events, particularly in gene promoter regions, are closely associated with the transcriptional regulation of stress-responsive genes [30]. Studies have revealed that tolerant varieties often exhibit more pronounced or more targeted methylation remodeling capacity [12]. In our study, salt-alkali stress triggered a decrease in CG and CHG methylation levels, while CHH methylation levels increased in *Medicago sativa*. These changes were particularly evident in promoter and transposable element (TE) regions (Fig. 1). This result aligns with previous findings that abiotic stresses frequently induce dynamic methylation reprogramming as a core epigenetic response. Specifically, promoter demethylation is often associated with transcriptional activation of stress-responsive genes, thereby contributing to enhanced stress tolerance [31]. The observed reduction in CG and CHG methylation may reflect the release of transcriptional repression, facilitating the expression of key defense-related genes under salt-alkali stress [32]. In contrast, the increase in CHH methylation is likely regulated by the RNA-directed DNA methylation (RdDM) pathway, which suppresses transposable element activity and maintains genome stability under stress conditions [33]. Notably, unlike commonly studied annual cereal crops such as rice and maize, Alfalfa must maintain tissue activity across growing seasons and endure multiple rounds of stress and recovery, which may increase its reliance on reversible regulatory mechanisms such as DNA methylation [34]. Moreover, breeding goals for forage crops focus on biomass and quality rather than seed yield. In this study, 5-azacytidine (5-AzaC) treatment not only enhanced salt-alkali tolerance but also significantly increased crude protein content and reduced ADF levels, suggesting that DNA methylation may participate not only in stress responses but also directly or indirectly influence forage quality—a phenomenon rarely observed in cereal crops [35].

**Fig. 7:**
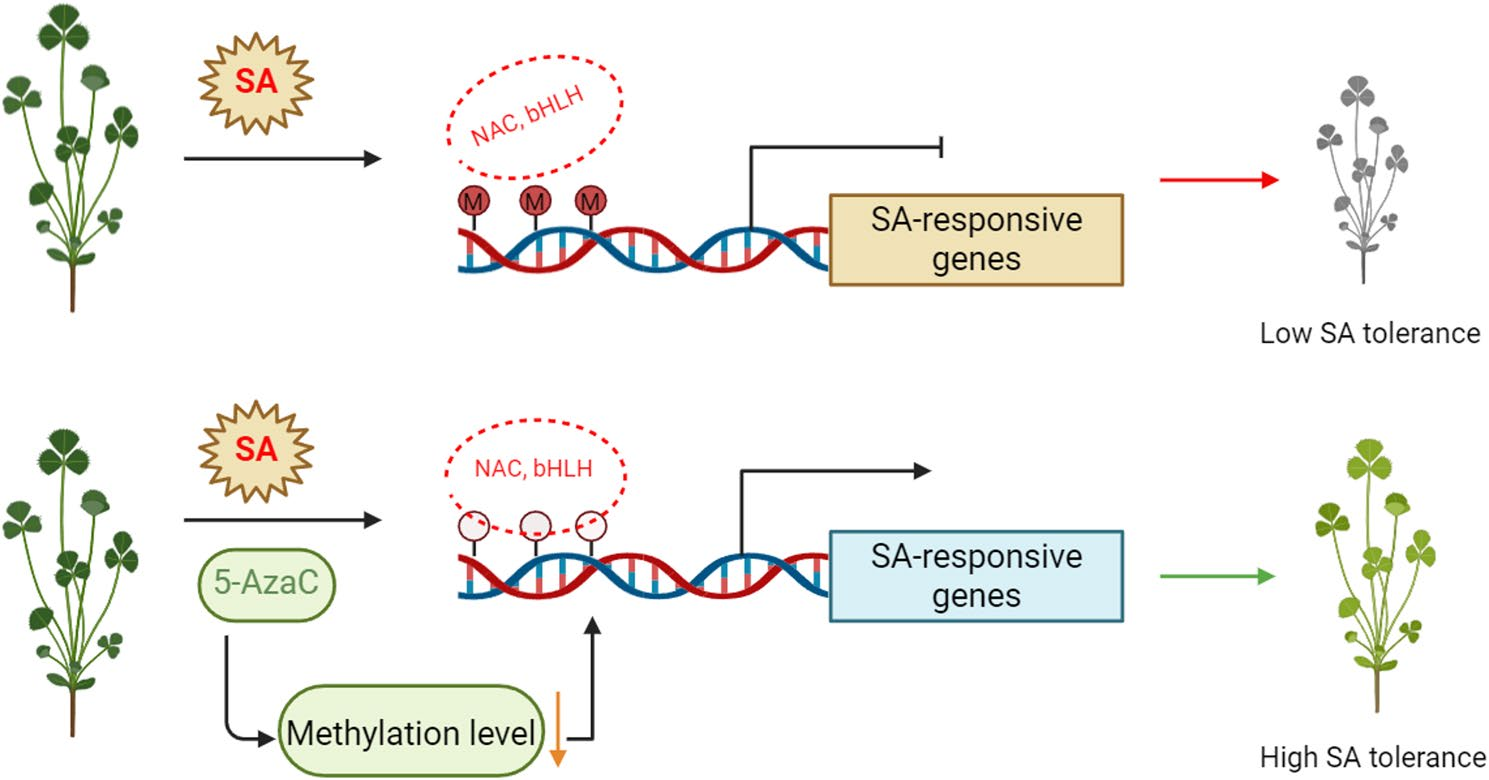
This diagram illustrates the hypothetical mechanism by which 5-AzaC enhances salt-alkali tolerance in alfalfa through DNA methylation regulation.

Further mechanistic analysis revealed that 5-AzaC treatment activated multiple stress-responsive metabolic pathways by upregulating a series of genes related to phenylpropanoid, flavonoid, and glutathione metabolism (Figs. 2, 3, 4 and 7). These pathways not only respond at the transcriptional level but also support enhanced stress tolerance at the physiological level. For example, upregulation of genes in the phenylpropanoid pathway, such as *PAL*, *4CL*, *COMT*, and *CAD*, facilitates accelerated lignin biosynthesis, strengthens cell walls, and enhances plant salt resistance [36]. Flavonoids act as non-enzymatic antioxidants, scavenging ROS and participating in signaling regulation, with some compounds potentially modulating the composition of the rhizosphere microbiome [37]. The glutathione metabolic pathway helps maintain redox homeostasis by sustaining the GSH/GSSG balance and AsA-GSH cycle function, thereby mitigating stress-induced damage [38]. The coordinated activation of these metabolic pathways is likely facilitated by enhanced transcription factor binding resulting from promoter demethylation, establishing a complete regulatory chain from DNA methylation changes to metabolic activation and physiological responses [39]. This regulatory pattern shares commonalities with mechanisms reported in other plants; for example, in strawberry, RdDM-mediated regulation of *quinone oxidoreductase* expression affects the accumulation of flavor compounds [40]; In apple, the DNA demethylase *ROS1* reduces promoter methylation of anthocyanin biosynthesis-related genes, enhancing their expression and promoting anthocyanin accumulation under cold stress [39]. DNA demethylation typically activates stress-responsive genes by releasing transcriptional repression, improving the accessibility of transcription factor binding sites (e.g., bZIP, MYB), and synergizing with histone deacetylation and demethylation to remodel chromatin into an open state [41, 42]. The promoter methylation changes observed in this study, along with the enhanced interactions with transcription factors such as NAC, bHLH, and ERF (Figs. 7 and 5), further support the above mechanism [43].

In summary, this study advances our understanding of the epigenetic regulatory mechanisms underlying alfalfa’s response to salt-alkali stress, highlighting the pivotal role of DNA methylation in abiotic stress adaptation. Notably, in *Medicago sativa*, a perennial forage crop, we observed that DNA methylation regulation affects both stress tolerance and forage quality, suggesting its potential as an important breeding target. Future research should focus on how DNA methylation modifications influence the binding efficiency of key transcription factors and the regulation of their target genes. By integrating functional genomics and metabolomics approaches, a systematic dissection of the epigenetic regulatory network can be achieved, providing a theoretical basis for the development of high-yielding, high-quality, and stress-resilient alfalfa varieties.

## Supplementary Information

Supplementary material 1.

Supplementary material 2.

## Data Availability

The data that support the findings of this study are openly available in National Center for Biotechnology at https://www.ncbi.nlm.nih. Previously published WGBS data are available under accession number PRJNA1211715. The RNA‑seq data generated in this study have been deposited in the NCBI Sequence Read Archive (SRA) under accession number PRJNA1284026.

## References

[1] Guo W, Chen J, Liu L, Ren Y, Guo R, Ding Y, Li Y, Chai J, Sun Y, Guo C. *MsMIOX2*, encoding a MsbZIP53-activated myo-inositol oxygenase, enhances saline–alkali stress tolerance by regulating cell wall pectin and hemicellulose biosynthesis in alfalfa. Plant J. 2024;120(3):998–1013. 39283985 10.1111/tpj.17032

[2] FAO. Global status of salt-affected soils. Rome: FAO; 2024.

[3] Crisp PA, Bhatnagar-Mathur P, Hundleby P, Godwin ID, Waterhouse PM, Hickey LT. Beyond the gene: epigenetic and cis-regulatory targets offer new breeding potential for the future. Curr Opin Biotechnol. 2022;73:88–94. 34348216 10.1016/j.copbio.2021.07.008

[4] Yung WS, Wang Q, Chan LY, Wang Z, Huang M, Li MW, Wong FL, Lam HM. DNA hypomethylation is one of the epigenetic mechanisms involved in Salt-Stress priming in soybean seedlings. Plant Cell Environ 2024 Nov.Epub ahead of print. 10.1111/pce.15297 39601237

[5] Hou X, Shi M, Zhang Z, Yao Y, Li Y, Li C, Yu W, Wang C, Liao W. DNA demethylation is involved in nitric oxide-induced flowering in tomato. J Integr Agric. 2025;24(5):1769–85.

[6] Tirnaz S, Batley J. DNA methylation: toward crop disease resistance improvement. Trends Plant Sci. 2019;24(12):1137–50. 31604599 10.1016/j.tplants.2019.08.007

[7] Zhang H, Lang Z, Zhu JK. Dynamics and function of DNA methylation in plants. Nat Rev Mol Cell Biol. 2018;19(8):489–506. 29784956 10.1038/s41580-018-0016-z

[8] Panda K, Mohanasundaram B, Gutierrez J, McLain L, Castillo SE, Sheng H, Casto A, Gratacós G, Chakrabarti A, Fahlgren N, et al. The plant response to high CO_2_ levels is heritable and orchestrated by DNA methylation. New Phytol. 2023;238(6):2427–39. 36918471 10.1111/nph.18876

[9] Zhao QQ, Lin RN, Li L, Chen S, He XJ. A methylated-DNA-binding complex required for plant development mediates transcriptional activation of promoter methylated genes. J Integr Plant Biol. 2019;61(2):120–39. 30589221 10.1111/jipb.12767

[10] Yao Y, Yang Y, Pan Y, Liu Z, Hou X, Li Y, Zhang H, Wang C, Liao W. Crucial roles of Trehalose and 5-azacytidine in alleviating salt stress in tomato: both synergistically and independently. Plant Physiol Biochem. 2023;203:108075. 37801738 10.1016/j.plaphy.2023.108075

[11] Feng SJ, Liu XS, Tao H, Tan SK, Chu SS, Oono Y, Zhang XD, Chen J, Yang ZM. Variation of DNA methylation patterns associated with gene expression in rice (*Oryza sativa*) exposed to cadmium. Plant Cell Environ. 2016;39(12):2629–49. 27412910 10.1111/pce.12793

[12] Wang L, Cao S, Wang P, Lu K, Song Q, Zhao FJ, Chen ZJ. DNA hypomethylation in tetraploid rice potentiates stress-responsive gene expression for salt tolerance. Proc Natl Acad Sci U S A. 2021;118(13):e2023981118. 33771925 10.1073/pnas.2023981118 PMC8020803

[13] Chen X, Chen G, Guo S, Wang Y, Sun J. *SlSAMS1* enhances salt tolerance through regulation DNA methylation of *SlGI* in tomato. Plant Sci. 2023;335:111808. 37482302 10.1016/j.plantsci.2023.111808

[14] Wang J, Zheng L, Peng Y, Lu Z, Zheng M, Wang Z, Liu J, He Y, Luo J. *ZmKTF1* promotes salt tolerance by mediating RNA-directed DNA methylation in maize. New Phytol. 2025;245(1):200–14. 39456131 10.1111/nph.20225

[15] Gao H, Xia X, An L. Critical roles of the activation of ethylene pathway genes mediated by DNA demethylation in *Arabidopsis* hyperhydricity. Plant Genome. 2022;15(2):e20202. 35319821 10.1002/tpg2.20202 PMC12807431

[16] Song X, Tang S, Liu H, Meng Y, Luo H, Wang B, Hou XL, Yan B, Yang C, Guo Z et al. Inheritance of acquired adaptive cold tolerance in rice through DNA methylation. Cell .2025;188(16):4213-4224. 10.1016/j.cell.2025.04.036 40409269

[17] Guo W, Sun Y, Chai J, Liu L, Li J, Ren Y, Guo C. *MsAREB1* enhances combined cold and saline–alkali stress tolerance by promoting ascorbic acid biosynthesis in alfalfa. Plant Biotechnol J. 2025;23(8):3349–62. 40415591 10.1111/pbi.70156 PMC12310818

[18] Li M, Yu A, Sun Y, Hu Q, Kang J, Chen L, Zhu X, Yang Q, Long R. Lipid composition remodeling and storage lipid conversion play a critical role in salt tolerance in alfalfa (*Medicago sativa* L.) leaves. Environ Exp Bot. 2023;205:105144.

[19] Arnon DI. Copper enzymes in isolated chloroplasts. Polyphenoloxidase in *Beta vulgaris*. Plant Physiol. 1949;24(1):1–15. 16654194 10.1104/pp.24.1.1 PMC437905

[20] Zhao J, Huang R, Wang X, Ma C, Li M, Zhang Q. Effects of combined nitrogen and phosphorus application on protein fractions and nonstructural carbohydrate of alfalfa. Front Plant Sci. 2023;14:1124664. 36968423 10.3389/fpls.2023.1124664 PMC10032370

[21] Mei H, Yan J, Jia X, Wang W, Li S, Sun R, Jiang H, Xie L, Zhou C, Bai S, et al. Transcriptomic and physiological analyses reveal that cytokinin is involved in the compound leaf development of alfalfa. Front Plant Sci. 2025;16:1460205. 39944174 10.3389/fpls.2025.1460205 PMC11814202

[22] Shi Y, Zhang Y, Yao H, Wu J, Sun H, Gong H. Silicon improves seed germination and alleviates oxidative stress of bud seedlings in tomato under water deficit stress. Plant Physiol Biochem. 2014;78:27–36. 24607576 10.1016/j.plaphy.2014.02.009

[23] Sheikh Mohammadi MH, Etemadi N, Arab MM, Aalifar M, Arab M, Pessarakli M. Molecular and physiological responses of Iranian perennial ryegrass as affected by trinexapac ethyl, Paclobutrazol and abscisic acid under drought stress. Plant Physiol Biochem. 2017;111:129–43. 27915174 10.1016/j.plaphy.2016.11.014

[24] Love M, Huber W, Anders S. Moderated Estimation of fold change and dispersion for RNA-Seq data with DESeq2. Genome Biol. 2014;15:550. 25516281 10.1186/s13059-014-0550-8 PMC4302049

[25] Yu G, Wang L-G, Han Y, He QY. ClusterProfiler: an R package for comparing biological themes among gene clusters. OMICS. 2012;16(5):284–7. 22455463 10.1089/omi.2011.0118 PMC3339379

[26] Gao R, Chen F, Chen L, Ma H. CHH Hypomethylation in Promoters of Oxidoreductase Genes May Contribute to Salt-Alkali Tolerance in Alfalfa (Medicago sativa L.). Plant Cell Environ. 2025. 10.1111/pce.70192. Epub ahead of print. PMID: 40963196. 10.1111/pce.70192 40963196

[27] Liu L, Si L, Zhang L, Guo R, Wang R, Dong H, Guo C. Metabolomics and transcriptomics analysis revealed the response mechanism of alfalfa to combined cold and saline-alkali stress. Plant J. 2024;119(4):1900–19. 38943631 10.1111/tpj.16896

[28] Liu Y, Wang J, Liu B, Xu Z-Y. Dynamic regulation of DNA methylation and histone modifications in response to abiotic stresses in plants. J Integr Plant Biol. 2022;64(12):2252–74. 36149776 10.1111/jipb.13368

[29] Sow MD, Le Gac AL, Fichot R, Lanciano S, Delaunay A, Le Jan I, Lesage-Descauses M-C, Citerne S, Caius J, Brunaud V, et al. RNAi suppression of DNA methylation affects the drought stress response and genome integrity in Transgenic Poplar. New Phytol. 2021;232(1):80–97. 34128549 10.1111/nph.17555

[30] Shi J, Xu J, Chen YE, Li JS, Cui Y, Shen L, Li JJ, Li W. The concurrence of DNA methylation and demethylation is associated with transcription regulation. Nat Commun. 2021;12(1):5285. 34489442 10.1038/s41467-021-25521-7 PMC8421433

[31] Tang M, Xu L, Wang Y, Dong J, Zhang X, Wang K, Ying J, Li C, Liu L. Melatonin-induced DNA demethylation of metal transporters and antioxidant genes alleviates lead stress in radish plants. Hortic Res. 2021;8:124. 34059663 10.1038/s41438-021-00561-8 PMC8167184

[32] Liu C, Li N, Lu Z, Sun Q, Pang X, Xiang X, Deng C, Xiong Z, Shu K, Yang F, et al. CG and CHG methylation contribute to the transcriptional control of OsPRR37-Output genes in rice. Front Plant Sci. 2022;13:839457. 35242159 10.3389/fpls.2022.839457 PMC8885545

[33] He L, Zhao C, Zhang Q, Zinta G, Wang D, Lozano-Durán R, Zhu J-K. Pathway conversion enables a double-lock mechanism to maintain DNA methylation and genome stability. Proc Natl Acad Sci U S A. 2021;118(35):e2107320118. 34453006 10.1073/pnas.2107320118 PMC8536323

[34] Zhou J, Xiao L, Huang R, Song F, Li L, Li P, Fang Y, Lu W, Lv C, Quan M, Zhang D, Du Q. Local diversity of drought resistance and resilience in Populus tomentosa correlates with the variation of DNA methylation. Plant Cell Environ. 2023;46(2):479–97. 36385613 10.1111/pce.14490

[35] Zhao T, Guan X, Hu Y, Zhang Z, Yang H, Shi X, Han J, Mei H, Wang L, Shao L, Wu H, Chen Q, Zhao Y, Pan J, Hao Y, Dong Z, Long X, Deng Q, Zhao S, Zhang M, Zhu Y, Ma X, Chen Z, Deng Y, Si Z, Li X, Zhang T, Gu F, Gu X, Fang L. Population-wide DNA methylation polymorphisms at single-nucleotide resolution in 207 cotton accessions reveal epigenomic contributions to complex traits. Cell Res. 2024;34(12):859–72. 39420233 10.1038/s41422-024-01027-x PMC11615300

[36] Rai A, Skårn MN, Elameen A, Tengs T, Amundsen MR, Bjorå OS, Haugland LK, Yakovlev IA, Brurberg MB, Thorstensen T. CRISPR-Cas9-mediated deletions of *FvMYB46* in Fragaria Vesca reveal its role in regulation of fruit set and phenylpropanoid biosynthesis. BMC Plant Biol. 2025;25(1):256. 40000946 10.1186/s12870-024-06041-0 PMC11853751

[37] Zhang C, Lu X, Yan H, Gong M, Wang W, Chen B, Ma S, Li S. Nitrogen application regulates antioxidant capacity and flavonoid metabolism, especially quercetin, in grape seedlings under salt stress. J Integr Agric. 2024;23(12):4074–92.

[38] Gul N, Ahmad P, Wani TA, Tyagi A, Aslam S. Glutathione improves low temperature stress tolerance in Pusa sheetal cultivar of *Solanum lycopersicum*. Sci Rep. 2022;12(1):12548. 35869119 10.1038/s41598-022-16440-8 PMC9307597

[39] Yu L, Sun Y, Zhang X, Chen M, Wu T, Zhang J, Xing Y, Tian J, Yao Y. *ROS1* promotes low temperature-induced anthocyanin accumulation in Apple by demethylating the promoter of anthocyanin-associated genes. Hortic Res. 2022;9:uhac007. 35147161 10.1093/hr/uhac007 PMC9123231

[40] Li Y, Shi Y, Li Y, Lu J, Sun Y, Zhang Y, Chen W, Yang X, Grierson D, Lang Z, et al. DNA methylation mediated by RdDM pathway and demethylation affects furanone accumulation through regulation of QUINONE OXIDOREDUCTASE in strawberry. Hortic Res. 2023;10(8):131. 10.1093/hr/uhad131 PMC10407599 37560014

[41] Zhu H, Wang G, Qian J. Transcription factors as readers and effectors of DNA methylation. Nat Rev Genet. 2016;17(9):551–65. 27479905 10.1038/nrg.2016.83 PMC5559737

[42] Long J, Liu J, Xia A, Springer NM, He Y. Maize decrease in DNA methylation 1 targets RNA-directed DNA methylation on active chromatin. Plant Cell. 2021;33(7):2183–96. 33779761 10.1093/plcell/koab098 PMC8364229

[43] Rimoldi M, Wang N, Zhang J, Villar D, Odom DT, Taipale J, Flicek P, Roller M: DNA methylation patterns of transcription factor binding regions characterize their functional and evolutionary contexts. Genome Biol 2024, 25(1): 146. 10.1186/s13059-024-03218-6 PMC11155190 38844976

