# Supplementary material for: DNA methylation changes in Medicago sativa under salt-alkaline stress and the function of 5-azacytidine in enhancing stress tolerance
Source: BMC Plant Biol. 2025 Oct 21;25:1419. doi: 10.1186/s12870-025-07424-7 (PMC12538822; doi:10.1186/s12870-025-07424-7)
Supplement: Supplementary file 2 — Supplementary material 2. [file 12870_2025_7424_MOESM2_ESM.docx]

Figure S1. Principal component analysis (PCA) of samples under different treatments.
